# Capping Exposed Pulps

**Published:** 1869-07

**Authors:** A. O. Rawls


### CAPPING EXPOSED PULPS.

BY A. O. RAWLS.

[Read before the Indiana State Dental Association.]

The delicacy of this operation must be apparent if we but note the fact that the Dental pulp is one among the most highly organized structures of our body, and responds to morbid influence through the medium of the most sensitive nerve of the entire nervous system. Besides the difficulties arising out of those conditions, it is enclosed within a wall of solid, unyielding bone, the resistance of which would prove quite an impediment to success, should the operation be performed in a rude, bungling manner, or at a time when inflammation was too great to admit of the probability of its being overcome in the natural way of vital resistance and recuperation. Viewing the subject in the light of other days, when the practice of capping an exposed nerve was in its incipiency, can we be surprised at the limited success met with and the meager support it received at the hands of our profession then, when to-day, with a theoretical and practical experience of twenty or thirty years in advance, and many valuable improvements to render us assistance, we fail in not a few of such cases intrusted to our care. Indeed, quite a number of the profession have abandoned the operation to considerable extent, resorting to it only when the pulp presents unmistakable signs of freedom from morbid conditions, while upon the other hand a few have turned their attention to therapeutical treatment when necessary, and, judging from the amount of success obtained in a comparatively short time, we would at least consider the practice commendable and well worthy a thorough trial.

When the practice of capping, for the purpose of protecting an exposed pulp first began to attract attention, its enemies were numerous and for several years the reign of arsenic or its kindred preparations continued unabated, but now we may rejoice in the thought that this fell destroyer has seen its palmiest days, and the possibility of saving an exposed pulp, when there exists but little inflammation, is no longer a question at issue, the only question being one as regards the relative value of the materials in use and the most satisfactory mode of manipulating the same to secure the best possible results.

If I mistake not, capping an exposed nerve or pulp dates prior to the operation of destroying it, and the first material used was the charred surface of the pulp itself, the actual cautery being used to produce the char, and this broken down tissue left remaining as a shield or barrier between the living pulp beneath and external filling, as might be inferred from the rudeness of the means resorted to and the nature of the parts involved, its use was not long continued; but the ill-success of this first attempt to fill over an exposed pulp, in all probability gave rise to the employment of means for its entire destruction. Shortly after this, metallic capping merged into use, sheet gold taking precedent, though on account of its conducting properties, soon yielded its laurels to lead and other materials of less heat-conducting powers, all of which have gradually fallen into disrepute; lead from its ease of adaptation to the wall of the cavity, and from the supposition entertained at one time that the oxyd deposited beneath the capping proved beneficial in allaying inflammatory action, has enjoyed quite an extensive reputation. In the mean time, chemical science has not failed to appreciate the difficulties of our position, or been derelict of her duty, but has advanced nobly to our assistance, and presents a material for our consideration which bids fair to eclipse all of its predecessors, and already opens a new era in the capping of exposed pulps. Its composition is chloride of zinc, in solution and calcined oxyd of zinc; and, I believe, the credit of first using this article as a filling for decayed teeth is due to Drs. Keep, of Boston, and Metcalf, of New Haven. Since then, not unlike other articles of merit, it has come very gradually into general use, improving in quality as its deficiencies were ascertained and the demand more extensive, until to-day it occupies a position enviable indeed, standing upon its own merits an auxiliary in operative Dentistry worthy of our esteem and recommendation. As a protective shield for an exposed pulp it has not been in general use many years, though for complete fillings and other purposes in which it has rendered valuable services, it has withstood a fair test for a considerable time.

All materials employed, or that have been in general use, and every theory linked with practical application in the Dental catalogue, has been burdened more or less with imperfections and objections, and, as a matter of course, oxy-chloride of zinc has its complete share, and if we were to judge and be governed by the opinions of a few, it certainly has an overdose.

Prominent among the objections urged against the use of this article as a shield over an exposed pulp is, first, that it is entirely too porous, consequently, when in close proximity to the pulp, would have a tendency toward absorbing all poisonous or effete matter existing at the point of contact, thereby rendering it unfit to be placed in such near relation with living tissues, laden as it would be with such impurities; second, that the escharotic properties possessed by the chloride is dangerous to the life of the pulp, and many cases are cited in which its use (rather abuse) has destroyed the life of this valuable structure. There are other objections, but those which I have noted seem to be the principle ones against its employment in this direction. As to the first mentioned, it is only necessary to state that our endeavor should be in the preparation of such cases to rid, if possible, the pulp and entire decayed cavity of the least indication of disorganized tissue or any like impurities. There should none form after the operation, the difficulty is overcome. To the second objection we would reply that a judicious use of the os-artificial, when well prepared, would obviate all such results, as the chloride is not taken into the circulation, and it is hardly probable that its use would destroy the pulp, unless employed in such quantities as to produce a great amount of inflammation.

The manner of introducing this material, and its consistency at the time it is introduced, tends as much probably to govern the results of the operation as any thing else concerned, and is, no doubt, too often overlooked or entirely disregarded, and failures from such neglect are credited to the material.

Should it be mixed too thick or allowed to dry out too much before introducing, the force required to adapt it closely to the walls of the cavity would give rise to congestion and consequent inflammation, or if placed in gently while thick as before, then there would exist a lack of cohesion in the particles of the filling; also, imperfect adaptation to the exposed surface of the pulp, the result of which would be crumbling of the cap upon introduction of the filling over it, or a place left between the shield and pulp, which condition would surely induce strangulation and death of the part involved, while a reverse of this mixing and introducing it of too thin a consistency would prove equally disastrous. We are all aware that a solution of chloride of zinc enters into the composition of os-artificial, and that it is endowed with powerful escharotic properties, and in case we should incorporate this substance too freely with the calcined oxyd, its effects would not only be very powerful, but would tend toward the production of no small amount of irritation, and probably to such an extent that the vital forces would not suffice to re-establish healthy action. We will grant, however, the possibility of there being sufficient reaction of the recuperative powers to counteract the irritation existing, in which event we have left for our consideration a thoroughly charred surface of the pulp at the point of exposure. The question now arises as to the probability of the char remaining *in situ*. If such were the case we would apprehend no danger whatever, though I am inclined to the opposite opinion that such is not the condition of affairs, but that the char is removed by absorption, not taken up by the capping material, though through the medium of the absorbent vessels of the pulp stimulated to increased action as a consequence of great irritation, thus ridding itself of the cause and leaving an intervening space between the filling and pulp, corresponding in size to the extent of broken down tissue, thereby rendering the possibility of success doubtful, as the space could not certainly exist without more or less trouble. However, this neglect should not argue against the usefulness of the material in such operations, but only guard us against its abuse. As regards my manner of introducing the oxy-chloride of zinc over an exposed pulp, I have nothing new to offer in that direction, and in conclusion would say that this material, when properly prepared and manipulated with the care that the delicacy of the operation requires is, in the vast majority of cases, far superior to any other article extant as a protection for exposed pulps or sensitive dentine, and especially is it invaluable as an additional shield between the filling and nerve, when there exists but a thin lamina of dentine over the latter.

